# Cost-effectiveness of influenza vaccine strategies for the elderly in South Korea

**DOI:** 10.1371/journal.pone.0209643

**Published:** 2019-01-25

**Authors:** Jae-Won Yun, Min Joo Choi, Gyeong-Seon Shin, Jae-Ok Lim, Ji Yun Noh, Yun-Kyung Kim, Joon Young Song, Woo Joo Kim, Sang-Eun Choi, Hee Jin Cheong

**Affiliations:** 1 Division of Infectious Diseases, Department of Internal Medicine, Guro Hospital, Korea University College of Medicine, Seoul, Korea; 2 Korea University College of Pharmacy, Sejong, Korea; 3 Department of Pediatrics, Ansan Hospital, Korea University College of Medicine, Ansan, Korea; Universidad Nacional de la Plata, ARGENTINA

## Abstract

**Objectives:**

Despite a high vaccine uptake rate of over 80% in South Korea, the disease burden of influenza is still high among the elderly, which may be due to low effectiveness of vaccines. Therefore, the cost-effectiveness of use among the elderly was analyzed in order to compare the current trivalent influenza vaccine (TIV) with a quadrivalent influenza vaccine (QIV) or MF59-adjuvanted trivalent influenza vaccine (ATIV).

**Methods:**

A static lifetime Markov model was used. It was assumed that the model would be repeated until individuals reached the age of 100. Cost-effectiveness was analyzed across three age groups (65–74 years, 75–84 years, and ≥85 years), and the at-risk group was studied.

**Results:**

Compared to the TIV, the QIV was expected to reduce the number of influenza infections by 342,873, complications by 17,011, hospitalizations by 8,568, and deaths by 2,031. The QIV was highly cost-effective when compared to the TIV, with a base case incremental cost-effectiveness ratio (ICER) estimated at USD 17,699/QALY (1USD = 1,151KRW), and the ICER decreased with age and was USD 3,431/QALY in the group aged 85 and above. Sensitivity analysis revealed that the ICER was sensitive to the QIV price, the proportion of influenza B, and vaccine mismatching. On the other hand, the ATIV was expected to reduce the number of influenza cases and complications by 1,812,395 and 89,747, respectively, annually, yielding cost-saving among all ages. ATIV price and vaccine efficacy were the most influential parameters for the ICER of ATIV.

**Conclusions:**

The QIV and ATIV strategies were considered more cost-effective in comparison to the TIV for vaccination strategies implemented for the elderly. However, owing to a lack of data on the effectiveness of ATIV among the elderly, a large-scale effectiveness study is required.

## 1. Introduction

Influenza is a viral respiratory disease occurring globally, causing socioeconomic losses by hindering healthy adults from working, and raising mortality and morbidity rates in high-risk groups. In South Korea, approximately 7,000 patients are hospitalized and 400,000 are treated as outpatients annually during winters, for seasonal influenza. However, during a pandemic, such as the H1N1 that occurred in 2009, more than 10 times the normal number of patients was reported [1].

To reduce the disease burden of influenza, the government has recommended vaccination for the target groups, including children under 18, elderly people aged 50 and above, pregnant women, and those with chronic diseases. Since 2005, free TIV has been provided for the elderly aged above 65 by the National Immunization Program (NIP). In addition, after the NIP was extended to private medical institutions in the 2015–16 influenza season the vaccination rate among elderly people aged 65 and above exceeded 80% [2, 3].

Despite the successful implementation of the influenza NIP, more than 2,900 deaths occur annually due to influenza, with most of them being elderly [4]. In order to reduce the disease burden of influenza among the elderly, the first aspect to consider is the problem of low vaccine efficacy among the elderly [5]. Therefore, highly immunogenic influenza vaccines, such as high-dose vaccine, MF59-adjuvanted vaccine, ASO3-adjuvanted vaccine, and intradermal vaccine, have been developed. Among these, only MF59-adjuvanted influenza vaccine (ATIV) has been approved in Korea since 2009. It has been used in approximately 5% of the NIP recipients since the 2015–16 influenza season.

The second issue in reducing the disease burden among the elderly is the problem of vaccine mismatch. Since 2000, two strains of influenza B have circulated concurrently, and concerns about the efficacy of the trivalent vaccine have been raised [6]. There are two lineages of the influenza B virus, namely, Victoria and Yamagata. However, only one lineage is included in the trivalent influenza vaccine (TIV) as recommended by the World Health Organization (WHO). As both lineages circulate simultaneously, vaccine mismatch is the foremost problem. In South Korea, the degree of influenza B mismatch was estimated to be 41.7% based on analysis during four influenza seasons (2007–08, 2009–10, 2011–12, and 2013–14). To overcome this problem, a quadrivalent influenza vaccine (QIV) was developed. In South Korea, people can receive either TIV or QIV based on their preferences, since 2014.

However, despite the introduction of the QIV and ATIV for the elderly aged 65 and above, most of them receive the TIV as the government subsidizes TIV as part of the NIP. Unless QIV and ATIV are included under the ambit of the NIP, the elderly may continue to receive the TIV. Therefore, we studied whether it would be cost-effective to replace TIV with either QIV or ATIV in the NIP for the elderly aged 65 and above. In addition, to confirm priorities due to the limited national budget, we conducted a subgroup analysis based on age and underlying medical conditions.

## 2. Methods

### 2.1 Model design and structure

**Fig 1:**
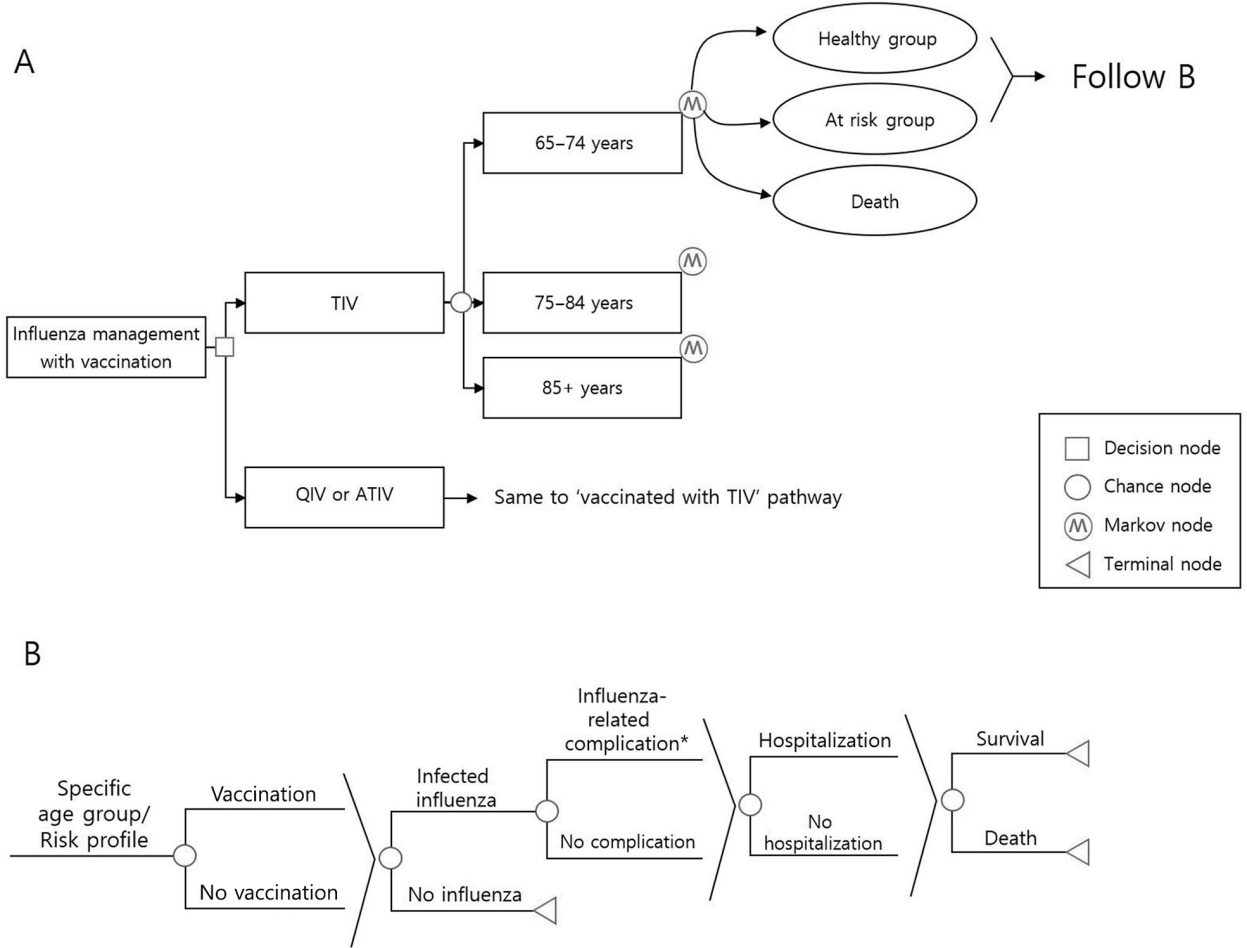
**A: Overview of Markov model structure; B: Overview of event pathways.** QIV, Quadrivalent influenza vaccine; TIV, Trivalent influenza vaccine; ATIV, Adjuvanted influenza vaccine. *acute complications or acute exacerbation of chronic diseases.

A static lifetime Markov model with a 1-year cycle time was used, and it was assumed that individuals aged 65 and older enter the model. At time zero in the year of 2016, South Korea population aged 65 and above was derived from Korean statistical information service, and stratified into three distinct age cohorts [7]. Three cohorts of different age groups (65–74, 75–84, 85 and older) enter the simulation, and the population ages one year for each cycle of the model. This Markov model includes three health states, namely, the healthy group state, at- risk group state, and death state. Individuals can stay in a state or move to another state once a year, until they reach age 100 or die (Fig 1A). It was assumed that the probabilities of moving from the healthy state to the at-risk state at individual age in every cycle are independent of vaccination status and influenza infection. The age-dependent probabilities were cited from foreign data due to the lack of domestic data [8]. In the annual state, a healthy or at-risk individual can proceed according to the probabilities of each event. We considered infections, complications, hospitalizations, and deaths as influenza-related events (Fig 1B). Post-exposure prophylaxis is excluded from the scope of interventions in the transition, considering the clinical situation, as they were rarely prescribed in South Korea (Figs 1A and 1B). Those who were infected but did not visit the clinic were excluded from the model. Survivors were forced to move on to the next annual transition. The base-case analysis compared QIV and ATIV with TIV over a lifetime time horizon. A subgroup analysis by age strata and underlying diseases was done. The results were presented as an incremental cost-effectiveness ratio (ICER) with a societal perspective. The discount rate was 3% for the base case analysis [9].

Subgroup analysis was conducted across three age groups (65–74 years, 75–84 years, and ≥85 years), and each age group was further divided into “healthy” and “at-risk” groups. The at-risk groups included those with any of the following, namely, chronic respiratory diseases, chronic heart diseases, chronic renal diseases, chronic liver diseases, neurological diseases, metabolic syndrome, malignancy, hematologic disease, or immunosuppressed states (S1 Table).

We used TreeAge Pro 2017 to develop the model and performed all the analyses.

### 2.2 Input data

#### Population data

The total population by age group was obtained from the Korean Statistical Information Service data [7], and the at-risk group was defined as those having outpatient/inpatient diagnostic codes corresponding to the group, within one year of the current influenza season (S1 Table).

#### Disease burden

The input data of the influenza disease burden was computed using the Korean National Health Insurance Service (NHIS) claims data [10] (Table 1). As almost all the citizens in Korea are enrolled to be part of the National Health Insurance, all data pertaining to medical services availed are registered in the insurance system, except medical treatments that are not covered by the National Health Insurance [11]. All medical records were available, including outpatient visits, inpatient records, and emergency room records. Admissions through the emergency room were classified as inpatient records, and visits to the emergency room were classified as outpatient visits. Using the diagnostic code (ICD-10), the incidence and hospitalization rates due to influenza in the population aged 65 and above were estimated for the 2013–14 and 2014–15 influenza seasons. Although the NHIS includes all medical service data, the disease burden could be underestimated if it were limited to influenza codes alone, as the codes are primarily confined to confirmed influenza cases alone. Therefore, in order to obtain adequate epidemiologic data due to influenza, we decided to extract the possible diagnostic codes while visiting a hospital with influenza-like illness (ILI). Influenza-related codes and ILI diagnostic codes were divided into four categories, namely, (1) acute upper respiratory disease, (2) bronchitis/bronchiolitis, (3) pneumonia, and (4) seasonal influenza (S1 Table). Based on the data of the ILI surveillance performed by the Korea Centers for Disease Control and Prevention (KCDC), the laboratory-confirmed rate of influenza was 0.392 among patients with ILI [12]. Therefore, considering the diagnostic codes corresponding to (1), (2), and (3) above, the number of cases was multiplied by 0.392. Subsequently, they were summed with the number of patients diagnosed with the diagnostic code for (4), to estimate the annual incidence of influenza.

**Table 1:** Input data for probability, cost, utilities in non-at-risk and at-risk groups.

Parameter	Range for Sensitivity Analysis			Ref
	Base	Low	High	
Population by age group				[7]
65–74 years	4,059,960	-	-	
75–84 years	2,440,081	-	-	
85+ years	632,385			
Probability				[10]
Influenza illness				
Non-at-risk group				
Flu case (seeking office visit)				
65–74 years	0.0502	0.0402	0.0602	
75–84 years	0.0543	0.0434	0.0652	
85+ years	0.0363	0.0290	0.0436	
Complication/Flu case				
65–74 years	0.0319	0.0255	0.0383	
75–84 years	0.0294	0.0235	0.0353	
85+ years	0.0397	0.0318	0.0476	
Hospitalization/Flu case				
65–74 years	0.0055	0.0044	0.0066	
75–84 years	0.0074	0.0059	0.0089	
85+ years	0.0137	0.0110	0.0164	
Mortality/Flu case				
65–74 years	0.0004	0.0003	0.0005	
75–84 years	0.0012	0.0010	0.0014	
85+ years	0.0073	0.0058	0.0088	
At-risk group				
Flu case (seeking office visit)				
65–74 years	0.1262	0.1010	0.1514	
75–84 years	0.1205	0.0964	0.1446	
85+ years	0.0964	0.0771	0.1157	
Complication/Flu case				
65–74 years	0.0778	0.0622	0.0934	
75–84 years	0.1026	0.0821	0.1231	
85+ years	0.1741	0.1393	0.2089	
Hospitalization/Flu case				
65–74 years	0.0503	0.0402	0.0604	
75–84 years	0.0786	0.0629	0.0943	
85+ years	0.1465	0.1172	0.1758	
Mortality/Flu case				
65–74 years	0.0059	0.0047	0.0071	
75–84 years	0.0158	0.0126	0.0190	
85+ years	0.0519	0.0415	0.0623	
Costs (USD)*				
Vaccination				
TIV	7.47	-	-	
QIV	8.59	7.47	11.56	Assumption
ATIV	8.59	7.47	11.56	Assumption
Vaccination administration cost	10.88	8.44	12.67	Government data
Outpatient visit^a^				[10]
Non-at-risk group				
65–74 years	13.73	10.98	16.48	
75–84 years	13.79	11.03	16.55	
85+ years	13.52	10.82	16.22	
At-risk group				
65–74 years	10.05	8.04	12.06	
75–84 years	8.88	7.10	10.66	
85+ years	7.50	6.00	9.00	
Outpatient visit with complication^a^				[10]
Non-at-risk group				
65–74 years	30.78	24.62	36.94	
75–84 years	31.99	25.59	38.39	
85+ years	34.36	27.49	41.23	
At-risk group				
65–74 years	34.13	27.30	40.96	
75–84 years	36.81	29.45	44.17	
85+ years	40.40	32.32	48.48	
Hospitalization^a^				[10]
Non-at-risk group				
65–74 years	1060.37	848.30	1272.44	
75–84 years	1054.50	843.60	1265.40	
85+ years	788.21	630.57	945.85	
At-risk group				
65–74 years	2375.08	1900.06	2850.10	
75–84 years	2156.13	1724.90	2587.36	
85+ years	1691.74	1353.39	2030.09	
Hospitalization with complication^a^				[10]
Non-at-risk group				
65–74 years	1107.07	885.66	1328.48	
75–84 years	1115.56	892.45	1338.67	
85+ years	1286.96	1029.57	1544.35	
At-risk group				
65–74 years	2820.24	2256.19	3384.29	
75–84 years	2713.84	2171.07	3256.61	
85+ years	2446.42	1957.14	2935.70	
Number of visits^a^				[10]
Outpatient visit				
Non-at-risk group				
65–74 years	1.41	1.13	1.69	
75–84 years	1.41	1.13	1.69	
85+ years	1.37	1.10	1.64	
At-risk group				
65–74 years	1.57	1.26	1.88	
75–84 years	1.56	1.25	1.87	
85+ years	1.50	1.20	1.80	
Outpatient visit with complication				
Non-at-risk group				
65–74 years	1.69	1.35	2.03	
75–84 years	1.65	1.32	1.98	
85+ years	1.53	1.22	1.84	
At-risk group				
65–74 years	1.77	1.42	2.12	
75–84 years	1.69	1.35	2.03	
85+ years	1.55	1.24	1.86	
Length of stay (days) ^a^				[10]
Hospitalization				
Non-at-risk group				
65–74 years	11.27	9.02	13.52	
75–84 years	10.66	8.53	12.79	
85+ years	9.40	7.52	11.28	
At-risk group				
65–74 years	14.73	11.78	17.68	
75–84 years	15.79	12.63	18.95	
85+ years	16.84	13.47	20.21	
Hospitalization with complication				
Non-at-risk group				
65–74 years	9.28	7.42	11.14	
75–84 years	9.11	7.29	10.93	
85+ years	10.28	8.22	12.34	
At-risk group				
65–74 years	14.01	11.21	16.81	
75–84 years	14.71	11.77	17.65	
85+ years	15.46	12.37	18.55	
Utilities^b^				
Baseline utility				
Non-at-risk group	0.867	0.690	1.000	[16]
At-risk group	0.737	0.680	0.890	[20,21]
Utility loss^c^				[17–19]
Outpatient care				
Without complication	0.35	0.28	0.42	
With complication	0.4	0.32	0.48	
Hospitalization				
Without complication	0.4	0.32	0.48	
With complication	0.5	0.40	0.60	
Discount rate (%)	3	0	5	[9]

^a^ Obtained from National Health Insurance Service claims data

^b^ Utiltiy is a scale of health related quality of life, where 1 means perfect health and 0 means being dead.

^c^ The duration of disutility was applied as 5 days for non-complicated outpatient influenza, 7 days for complicated outpatient influenza, and the total average length of stay for hospitalized influenza.

*1USD = 1,151KRW.

Owing to the lack of epidemiological data on the incidence of post-influenza complications, we estimated the acute complication rates and the acute exacerbation rates of chronic diseases that may occur after the diagnosis of influenza, using the NHIS claims database [10]. Acute complications included hospitalization or outpatient visits with complication diagnostic codes that occurred within four weeks of contracting influenza. However, in order to improve the association with influenza, it was limited to cases in which there was no medical history due to the same complication code within one year before the influenza occurred. Acute complications among the elderly included pneumonia, otitis media, sinusitis, encephalitis, myositis, myocarditis, pericarditis, acute myocardial infarction, stroke, rhabdomyolysis, and transverse myelitis (S1 Table). The acute exacerbation of a chronic disease was defined as hospitalization caused by a disease, such as chronic respiratory disease, chronic liver disease, renal failure, heart disease, and diabetes, within four weeks of influenza infection, only if admission due to the same code was not done within a year prior to the influenza event (S1 Table). It meant that the chronic disease was controlled well prior to the influenza episode.

Influenza-related mortality was defined as deaths within four weeks after the diagnosis of influenza. All-cause mortality rates in non-at-risk group were obtained from the Korea National Statistical Office (S2 Table) [7]. All-cause mortality rates in at-risk groups were assumed to be 1.3 times higher than non-at-risk groups.

#### Cost data

Using the NHIS claims data for 2013–14 and 2014–15 influenza seasons, we extracted the cost of the corresponding diagnostic code (ICD-10) as presented in Table 1 and S1 Table[10]. Medical expenses per person were calculated for the following four cases, namely, hospitalization for influenza, outpatient for influenza, hospitalization for influenza complications, and outpatient treatment for complications due to influenza. In the case of hospitalization costs, the entire billing cost was estimated. However, in order to exclude the possibility of additional costs other than diagnosis and treatment of influenza in the outpatient setting, medication costs were excluded from the outpatient service costs for the first two weeks after the diagnosis. Instead, we added influenza rapid antigen test costs (USD 17.38) and oseltamivir costs (USD 22.47). However, in the case of complications after the diagnosis of influenza, other drugs, such as antibiotics, are more likely to be prescribed, and are thus included in the total cost of claims as part of medication costs. Transportation costs were estimated using the Korea Health Panel data of 2008 [13], and the cost of caregivers was estimated using the Korea Health Panel data of 2013, but only applied during hospitalization [14]. All costs were adjusted for the prices of 2016, using the consumer price index of the Korean Statistical Information Service, taking inflation into account, and converting costs to US dollars based on the exchange rate in 2016 (1USD = 1,151KRW) [7].

We did not consider the loss in productivity due to the patient's time loss as an indirect cost. The elderly aged 65 and above, were assumed to have no income as people in Korea generally retire before 65.

The cost of the TIV was set at USD 7.47, which was the purchase price of the NIP in the 2016–17 season, and the price of the QIV and ATIV were assumed to be USD 8.59, a 15% increase over the TIV. The vaccination fee was USD 10.88, which was the same as the existing NIP.

#### Data on circulating influenza type or subtype

The proportions of influenza A and B were collected from the hospital-based influenza morbidity and mortality (HIMM) surveillance data from the 2011–12 influenza season to the 2015–16 season. Although the KCDC holds data from nationwide surveillance, we used the HIMM data. There is a possibility that the fraction of influenza B is overestimated in the KCDC data as a fixed number of cases are analyzed regardless of the epidemic size of each influenza type or subtype. The HIMM surveillance was implemented in ten tertiary hospitals distributed throughout the country since the 2011–12 influenza season and more details have been introduced in past literature [15]. A total of approximately 6,000 cases were analyzed. The fraction of influenza A and B were 79.9% and 24.3%, respectively (both A and B were 4.1% at the same time). The mismatching rate between circulating influenza B viruses and lineage of vaccine strains was 41.7%.

#### Utility data

Using solitary national utility data published in 2014, 0.867 was applied to those aged 65 and above [16]. As domestic studies on disutility due to influenza have not been conducted thus far, we determined the influenza-attributed disutility through expert meetings considering the foreign data and domestic medical environment [17–19] (Table 1). The duration of disutility was applied as 5 days for non-complicated outpatient influenza, 7 days for complicated outpatient influenza, and the total average length of stay for hospitalized influenza. In the expert meeting, we conducted a two-round modified Delphi survey with seven experts. In the case of at-risk patients with chronic diseases, utility can be expected to decrease in comparison to the case of the general population. However, as there were no domestic data, it was also determined through expert meetings based on the results of foreign studies. Considering the average illness severity of the at-risk group, we applied a 15% reduction in comparison to the utility of a healthy person [20, 21].

#### Vaccine characteristics

The vaccine efficacy in influenza A was assumed to be the same in the TIV and QIV [22] (Table 2). Vaccine efficacy against influenza B was cited from a meta-analysis perspective for adults in vaccine matching and mismatching influenza season [23]. We assumed that the trivalent vaccine had some cross-protection against the mismatched influenza B lineage following this meta-analysis. Among the elderly, as vaccine efficacy was lower in comparison to the efficacy in adult groups, vaccine efficacy for the elderly was adjusted. The vaccine efficacy ratio between adults and the elderly was based on the Cochrane Reviews [22, 24]. On the other hand, the efficacy of ATIV was determined as being 25% higher in comparison with the TIV considering the relative risk of influenza-related hospitalization from a cohort study [25] (Table 2). The vaccine efficacies for influenza A and B were assumed to be the same in at-risk groups. Vaccine-related adverse events were excluded from the model as these events were assumed equal for TIV, QIV and ATIV. Also, we assumed duration of immunity (vaccine-induced) as one year time for all three vaccine formulations. The vaccination rate of 80% was applied to the elderly aged 65 and above, considering that the coverage rate was 81% and 82% in the 2015–16 and 2016–17 influenza seasons, respectively [3]. The main analysis repeated vaccinations using the same assumptions every year until all patients in the cohort reach age 100 or die.

**Table 2:** Input data for vaccine efficacy in non-at-risk and at-risk groups.

		Influenza A		Influenza B		Ref
			match^a^	mismatch^b^	average^c^	
	Trivalent	58.00	57.00	36.00	48.24	[22–24]
**Vaccine efficacy, %**	Quadrivalent	58.00		57.00		[22–24]
	Adjuvant	72.50	71.25	45.00	60.30	[25]

^a^ When the viruses in the vaccine and the viruses circulating among people during a influenza season are closely related

^b^ When the circulating virus is different from the vaccine virus

^c^ (Vaccine efficacy in matched season × matching rate between circulating influenza B virus and lineage of vaccine strain) + (Vaccine efficacy in mismatched season × mismatching rate between circulating influenza B virus and lineage of vaccine strain).

### 2.3 Sensitivity analysis

One-way sensitivity analysis and probabilistic sensitivity analysis (PSA) were performed with all input parameters. Probabilities were assigned binomial distributions, medical costs excluding vaccination were assigned gamma distributions, and number of visit (length of stay) used normal distributions. Vaccination related cost and utilities were assigned triangular distributions. Considering the variation in influenza vaccine efficacy in each season, we also included a scenario where the intervention is only offered for 1 year in the sensitivity analysis. According to the recommendations of the WHO, an ICER below a per capita gross domestic product (GDP) is highly cost-effective, and an ICER less than three times the per capita GDP is considered cost-effective [26]. The per capita GDP for Korea was USD 35,751 in 2016 [27]. Therefore, we considered USD 35,751/Quality-adjusted life year (QALY) as a highly cost-effective threshold.

## 3. Results

The estimated number of individuals vaccinated and the lifetime disease burden (the estimated number of influenza cases seeking medical treatment, complications, hospitalization for influenza, or deaths) with the TIV, QIV, and ATIV are shown in Table 3.

**Table 3:** Expected clinical outcome of trivalent, quadrivalent, and adjuvanted influenza vaccines.

	TIV	QIV	ATIV	Diff (QIV–TIV)	Diff (ATIV–TIV)
Lifetime disease burden					
Number vaccinated	141,636,990	141,638614	141,645,515	1,624	8,525
Number of influenza cases (Seeking medical treatment)	11,335,153	10,992,280	9,522,758	-342,873	-1,812,395
Number with influenza complications	560,936	543,925	471,189	-17,011	-89,747
Number of hospitalizations for influenza	281,974	273,405	236,823	-8,568	-45,151
Number of influenza deaths	66,476	64,445	55,819	-2,031	-10,656
Life-years	173,287,259	173,309,862	173,310,920	22,603	23,661

TIV: Trivalent influenza vaccine; QIV: Quadrivalent influenza vaccine; ATIV: Adjuvanted trivalent influenza vaccine.

### 3.1 Base case analysis

The QIV would be expected to further reduce the number of influenza cases by 342,873, complications by 17,011, hospitalizations by 8,568, and deaths by 2,031 in comparison to the TIV (Table 3). Using QIV would result in 22,603 additional life years gained compared to the use of TIV. As a result, the QIV was highly cost-effective in comparison to the TIV, with an ICER estimated at USD 17,699/QALY (Table 4). ATIV is anticipated to reduce influenza by a significant extent. The reduction in the number of influenza cases and complications are expected to be 1,812,395 and 89,747, respectively. In addition, hospitalization and death were expected to be reduced by 45,151 and 10,656, respectively (Table 3). Using ATIV instead of TIV, 23,661 additional life years were gained. As a result, the ATIV showed that the cost reduction due to reduced influenza disease burden exceeded the cost increase due to the ATIV price, resulting in cost savings (dominant strategy) (Table 4).

**Table 4:** Base case analysis (per-person costs and effectiveness).

		Cost per person	Incremental cost	Effectiveness	Incremental effectiveness	Incremental cost-effectiveness ratio
		USD*		QALY		USD/QALY
Total (≥65 years)	TIV	346.34	-	14.04235	-	-
	QIV	353.48	7.14	14.04276	0.00040	17,699
	ATIV	334.58	-11.76	14.04445	0.00210	- 5,597

TIV: Trivalent influenza vaccine; QIV: Quadrivalent influenza vaccine; ATIV: Adjuvanted trivalent influenza vaccine; QALY: Quality-adjusted life years

*1USD = 1,151KRW

Results of subgroup analysis based on age and underlying medical conditions, the ICER for the QIV compared with the TIV decreased with age and was found to be USD 3,431/QALY in the group aged 85 and above (Table 5). The ICER in the at-risk group aged 65 and above was USD 1,064/QALY. On the other hand, the results for the ATIV compared the TIV showed cost savings (dominant strategy) in all age groups and at-risk groups (Table 5).

**Table 5:** Subgroup analysis based on age and medical conditions (per-person costs and effectiveness).

		Cost per person	Incremental cost	Effectiveness	Incremental effectiveness	Incremental cost-effectiveness ratio
		USD*		QALY		USD/QALY
65–74 years	TIV	380.59	-	17.09250	-	-
	QIV	389.76	9.17	17.09276	0.00037	24,897
	ATIV	370.91	-9.68	17.09431	0.00192	- 5,044
75–84 years	TIV	313.83	-	10.33998	-	-
	QIV	318.42	4.73	10.34042	0.00044	10,393
	ATIV	298.56	-15.27	10.34228	0.00230	- 6,644
≥85 years	TIV	210.52	-	5.43123	-	-
	QIV	212.31	1.79	5.43176	0.00052	3,431
	ATIV	197.12	-13.40	5.43396	0.00272	- 4,923
At-risk	TIV	526.04	-	11.91314	-	-
	QIV	526.87	0.83	11.91392	0.00078	1,064
	ATIV	483.59	-42.45	11.91721	0.00406	- 10,445

TIV: Trivalent influenza vaccine; QIV: Quadrivalent influenza vaccine; ATIV: Adjuvanted trivalent influenza vaccine; QALY: Quality-adjusted life years

*1USD = 1,151KRW.

### 3.2 Sensitivity analysis

**Fig 2:**
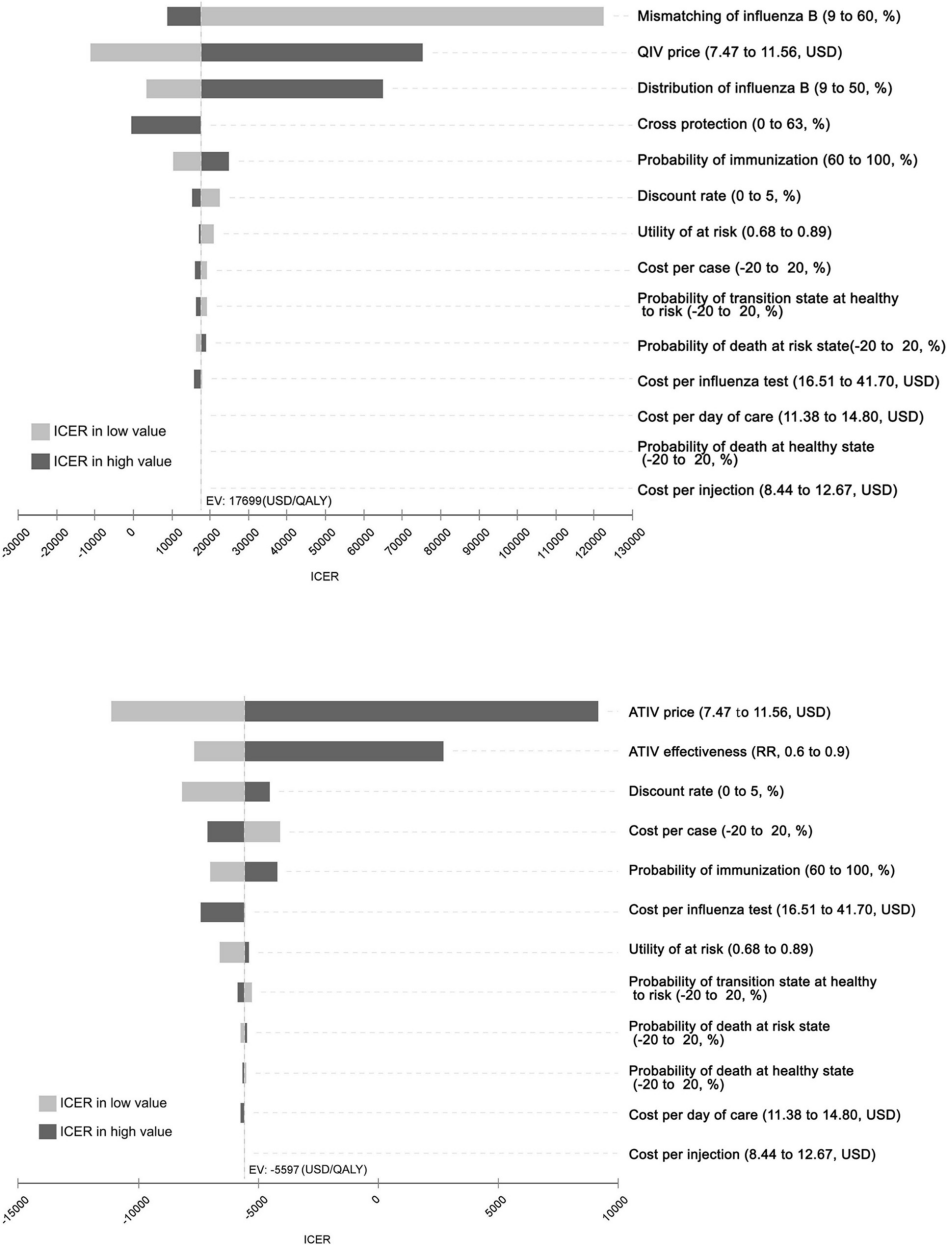
One-way sensitivity analysis. (A) Quadrivalent influenza vaccine (QIV) and (B) Adjuvanted trivalent influenza (ATIV) in the elderly aged 65 and above.

**Fig 3:**
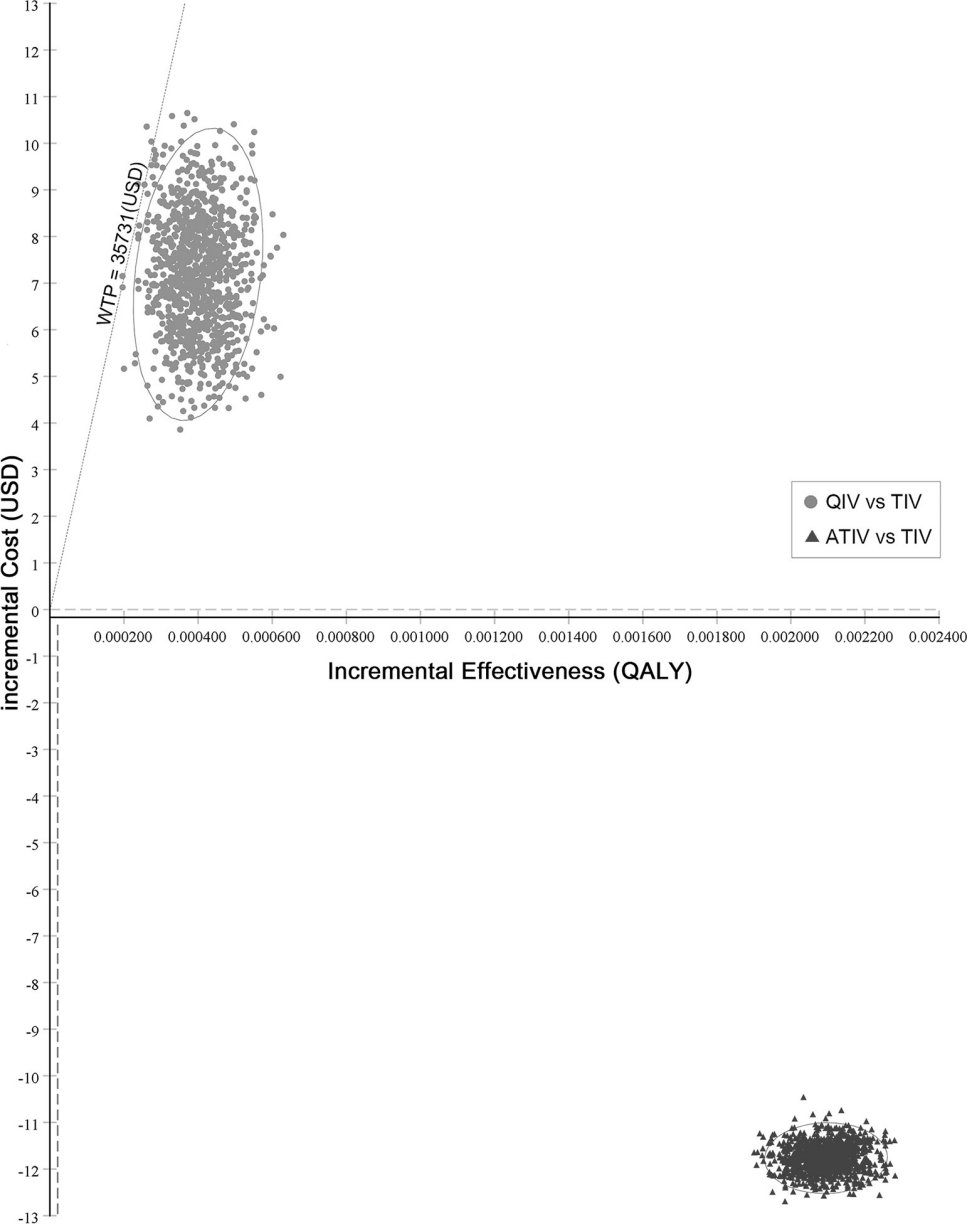
Probabilistic sensitivity analysis. Quadrivalent influenza vaccine (QIV was favored over 99% for willingness to pay (WTP) USD 35,731/QALY compared to TIV) and adjuvanted trivalent influenza vaccine (ATIV was cost-saving compared to TIV) among the elderly aged 65 and above. Willingness to pay; WTP.

Fig 2A shows the one-way sensitivity analysis of the QIV among the elderly. The ICER was sensitive to three parameters among the elderly, namely, the QIV price, proportion of influenza B, and mismatching between the TIV and circulating influenza B lineage. Among the three parameters, the degree of vaccine mismatch had the most substantial effect on the ICER among the elderly (Fig 2A). The QIV was more cost-effective in comparison to the TIV when the QIV price was less than 120% of the trivalent vaccine price, and the influenza B fraction was more than 20%, and the mismatch rate was more than 30%. PSA was performed and the QIV was favored over 99% who were willing to pay USD 35,731/QALY in comparison to the TIV (Fig 3).

Fig 2B shows the one-way sensitivity analysis of the ATIV among the elderly. In the case of ATIV, the parameters that affected the ICER the most were vaccine prices and vaccine efficacy (Fig 2B). Sensitivity analysis showed that ATIV was highly cost-effective at USD 9,155/QALY, even when the price of vaccines increased by 55% (USD 11.58) in comparison to the TIV, and the ICER continued to be cost-effective at USD 2,700/QALY, even when the relative risk of the ATIV was reduced to 0.9. The PSA result of the ATIV was that of a cost-saving (Fig 3).

Table 6 shows the results for the scenario analysis where the intervention is only offered for 1 year. A strategy of vaccinating the QIV for only 1 year resulted also favorable estimated cost-effectiveness (ICER USD 34,008/QALY) as like the base-case. A strategy of vaccinating the ATIV instead of TIV for only 1 year resulted also cost-saving (dominant strategy) (Table 6).

**Table 6:** The scenario analysis where the intervention is only offered for 1 year.

		Cost per person	Incremental cost	Effectiveness	Incremental effectiveness	Incremental cost-effectiveness ratio
		USD*		QALY		USD/QALY
Total (≥65 years)	TIV	346.34	-	14.04235	-	-
	QIV	346.98	0.64	14.04237	0.00002	34,008
	ATIV	345.92	-0.42	14.04245	0.00010	- 4,280

TIV: Trivalent influenza vaccine; QIV: Quadrivalent influenza vaccine; ATIV: Adjuvanted trivalent influenza vaccine; QALY: Quality-adjusted life years

*1USD = 1,151KRW.

## 4. Discussion

This study estimated the disease burden of influenza using NHIS claims data and analyzed the cost-effectiveness of the QIV and ATIV versus TIV in the NIP for the elderly. To determine the priority of vaccine replacement, we conducted a cost-effectiveness analysis (CEA) among the elderly, based on the age and underlying medical condition. This study showed that the new QIV and ATIV were very cost-effective in reducing the burden of influenza, complications, and the resulting burden of hospitalization and death despite the high prices. It was also found to be more cost-effective as age increased and was even more cost-effective in groups with chronic diseases.

In the model, we assumed that the prices of the QIV and ATIV are the same. Sensitivity analysis showed that the price of QIV was an important factor affecting the outcome. Currently, the NIP does not officially include the QIV; therefore, it was a challenging and important decision to set a price for the vaccine in the model. ATIV is usually more expensive than the QIV and TIV are, in a clinical setting, but when the ATIV (5% of the total) was partially included in the NIP during 2015–16 season, it was contracted at the same price of the TIV. Vaccines are generally procured at prices lower than the official prices. Therefore, if the QIV and ATIV are included in the NIP, ATIV would not be more expensive than QIV. Thus, the prices of both vaccines were set at 15% higher in comparison to the TIV in this study. However, if the price difference introduced in the actual NIP is large, the result may vary according to changes in vaccine prices. Nevertheless, the ICER value of the ATIV continued to be dominant in the sensitivity analysis, where the price of ATIV increased by 25% (USD 9.34) in comparison with the TIV. The ICER value of the QIV also continued to be cost-effective, where the price of QIV increased by 20% (USD 8.97).

Vaccine efficacy appears to have played an important role in the results in each model. Vaccine efficacy of the QIV varied according to the proportion of influenza B and vaccine mismatch, but its size of change was not large. On the other hand, as the efficacy of ATIV on influenza A and B was greater than that of the TIV, the ICER value (ATIV versus TIV) was dominant. To date, there have been no randomized controlled trials on the efficacy of the ATIV, and retrospective studies are also very limited. Therefore, if there are further studies on the efficacy of ATIV, the results could change. However, in the present study, the result of sensitivity analysis was very robust, and ATIV was still cost-effective compared to TIV when the vaccine efficacy of ATIV was applied as 10% higher than that of TIV (relative risk of 0.9).

In the CEA, vaccine-related adverse events were not considered. We assumed that the adverse effects were not greater in the QIV and ATIV groups when compared to TIV. Past meta-analysis has shown that compared to TIV, QIV had more common injection-site pain. However, there was no difference in the aggregated local and systemic adverse events between QIV and TIV [28]. On the other hand, according to a study by Villa M et al., adverse effects due to ATIV over 65 years did not show any significant difference from TIV [29]. Further, in the study of Frey SE et al., the rates of reactogenicity were higher in the ATIV group as opposed to the TIV group, but reactions were mostly mild-to-moderate and transient [30]. Therefore, we concluded that these differences are not a significant factor in affecting disutility or treatment cost between groups. As for the duration of vaccine-induced immunity, we assumed that protective immunity does not last more than one year. Recently, many studies have reported the waning of influenza vaccine immunity during the influenza season [31, 32]. With respect to the vaccine formulations, there is no clear data on the difference of duration for protective immunity, but the duration is not expected to exceed one year. A majority of studies in the past have shown favorable results for the QIV despite the differences in various input data [33]. Similar to past studies, the key parameters affecting cost-effectiveness outcomes in this study were mismatching of influenza B, the QIV price, distributions of influenza B, and cross-protection of the TIV against the influenza B virus. This study follows earlier studies in applying a mismatching rate of 41.7%, distribution of influenza B of 24.3%, and cross-protection of 63% in the base case analysis [33]. However, while past studies have shown a vaccine price difference between the TIV and QIV that ranged from $ 1.25 to $ 7.14, in this study, the difference in vaccine price was $ 1.12, which was small in comparison to what was found in other studies. Although the difference in the prices of the QIV and TIV of this study was not large in comparison to the difference noticed in past studies, the results were similar due to variations in other input data [34, 35].

The only study on the CEA of ATIV versus the TIV was conducted in Canada [36]. The Canadian study using dynamic modeling is not directly comparable to this study. However, the ICER was USD 2,111/QALY, and the ATIV was highly cost-effective over the TIV. Even though the price of ATIV was USD 4 higher than the TIV in the Canadian study, owing to other input data, including vaccine efficacy and difference in medical costs, the result was similar to the present study.

Although QIV and ATIV had equivalent vaccination coverage with TIV, immunization counts increased over the entire cycle due to lower mortality compared to TIV (Table 3). Owing to better vaccine efficacy in the QIV and ATIV groups, the survivors increased and the number of subjects requiring vaccination increased. This resulted in higher total immunization costs in the QIV and ATIV groups compared to TIV, but the results were cost-effective with reduced influenza infection and treatment costs.

This study has several limitations. First, several recent studies have published results derived from using dynamic models [37, 38]. However, this study did not apply the herd effect and transmission pattern as the model of study was limited to the elderly, rather than being extended to the whole population. However, the probability used in this study reflects the real situation and therefore can be stated that the indirect effect has already been applied. In addition, even if we assume that we use a dynamic model, it is expected that the results of this study will be more favorable to the QIV and ATIV, owing to the increased vaccine efficacy. In addition, as the vaccine coverage increases, the impact of the indirect effect is reduced [39]. In this study, we applied the 80% coverage rate to the model in consideration of the current vaccination rate to ensure that the additional effect due to the herd effect is relatively small.

Second, we could not sufficiently reflect the seasonal variability from NHIS claims data of only two influenza seasons, nor could we distinguish between the disease outcome and medical cost due to influenza A and influenza B infections. However, since 2011, except for the 2012–13 influenza season, the size of the epidemic was not significantly different [40], and a study in the past has shown that the clinical symptoms and outcomes of influenza A and B infections were similar [41]. Additionally, the results were robust even though variability was considered in the sensitivity analysis.

Third, the input data used may have marginally underestimated the disease burden. Patients with mild influenza might not visit hospitals, so they would be excluded from the NHIS claims data. The disease burden was likely to be rather reduced as the cost of over-the-counter drugs that they may have used was excluded. In addition, caregiver costs were applied conservatively in comparison to past studies [42]. However, the disease burden of mild influenza would not be large, and this also appears to support the results of this study if higher values are applied.

Finally, we did not apply the regression based method when calculating the treatment cost. However, in order to avoid overestimating costs attributable to influenza, we excluded almost all treatment costs except the primary care fees for influenza and treatment costs for influenza-related complications or hospitalization. Medical costs for chronically ill patients were included when they visited the out-patients clinic with influenza, but the treatment costs for the underlying disease itself were excluded. When calculated using this method, the cost of outpatient treatment was only USD 7 ~ 14 for non-complication and USD 30 ~ 40 for complication. Even after combining the costs of oseltamivir and rapid antigen test, the medical costs in this study were rather lower than those in a previous study in South Korea [42]. As for the foreign countries, outpatient treatment costs were estimated to be USD 30 ~ 170 and hospitalization costs were USD 1,900 ~ 10,251[8, 34, 35]. Although direct comparison with foreign data is limited, this study might not overestimate the medical costs.

Despite these limitations, this study has the advantage of using data that reflect the domestic reality. The question of whether the estimated incidence, complication rate, and hospital admission rate of the influenza using ILI, will correlate with the actual influenza burden over time would be important. Considering that the influenza disease burden might be underestimated using the laboratory-confirmed influenza ICD-10 codes (J09–J11) in comparison to the hospital-based surveillance data [1, 42], we included ILI codes along with the application of influenza proportion in this study. In this study, the incidence of influenza in the elderly aged 65 and above was approximately 6%, hospitalization rate was 4%, and mortality rate was 1%. The results are consistent with the probabilities obtained in past studies [34, 43]. Furthermore, in the community-based influenza study conducted by the Ministry of Health and Welfare, the influenza incidence of the elderly aged 65 and above in the 2013–14 influenza season was approximately 4% [44].

So far, several studies have been conducted to evaluate the influenza disease burden in South Korea. The contribution of the most recently published hospital-based study was that only laboratory-confirmed influenza cases were considered. However, as it was limited to a tertiary hospital, the disease burden of primary and secondary hospitals was excluded. In addition, it is possible that the medical cost per person could have been overestimated as the illness severity in case of hospitalization in the tertiary hospital was high [42]. This study addressed these limitations and showed how to the measure actual influenza disease burden using NHIS claims data more accurately. More importantly, we could measure the actual disease burden (incidence, complications, and medical costs) among the elderly, stratified by underlying medical conditions and age groups. The results of the analysis came out as we expected, except for the length of stay (LOS) for complicated influenza. It is unclear why LOS for complicated influenza is shorter than that for non-complicated cases. However, this is probably due to the strict case definition for hospitalized complicated influenza. We have defined influenza-related acute complication as a case that has never been admitted with the same code within a year before the influenza outbreak. We used this definition to distinguish complications due to influenza from the worsening of uncontrolled underlying disease. However, if a patient with chronic disease is exacerbated by influenza, the patient may fall into a non-complicated group, which can lead to misclassification. We used this method to conservatively set the burden of disease caused by influenza, inevitably leading to overestimation of LOS for non-complicated cases. Nevertheless, this would not affect the cost-effectiveness results because such a misclassification is applied to each vaccine in the same manner.

In addition, the elderly with chronic diseases are expected to have a higher disease burden in comparison to the healthy elderly, but their data have not been reported thus far in South Korea. We estimated the disease burden related influenza of the 'at-risk group' using the diagnostic code corresponding to the chronic disease (S1 Table). As most of the diseases we defined as 'at-risk group' are diseases that are difficult to misdiagnose, we defined 'more than one physician claim' as the case definition. Of the total patients, 19.5% were at-risk group and we agreed that the case definition was not overestimated (data not shown). In this study, the incidences and complications of influenza among the elderly with chronic diseases were more than twice of those who were healthy, and the hospitalization and mortality rates were ten times more. Treatment costs were not significantly different between the at-risk group and the healthy elderly group in the outpatient treatment setting, but it increased more than twice among the at-risk group when hospitalized. Therefore, if the health budget is limited, it may be warranted to vaccinate the QIV or ATIV for seniors with chronic diseases, thereby reducing the entire disease burden.

In conclusion, this study showed that switching from the TIV to QIV or ATIV among the elderly would be cost-effective in the domestic situation if the vaccination rate of more than 80% is maintained in South Korea. Despite the recent controversy over the criteria of WHO’s cost-effectiveness thresholds [45, 46], this study suggests that vaccination replacement needs to be considered, as the ICER of the result is much lower in comparison to the GDP values or cost savings. However, to determine which vaccine is optimal for the elderly, a CEA between the QIV and ATIV is required. Moreover, large-scale studies are required to better evaluate the effectiveness of ATIV. In addition, as high-dose vaccines have not been introduced in Korea yet, it is necessary to study the comparative vaccine effectiveness and CEA among the QIV, high-dose vaccines, and ATIV when all these vaccines are available in the future.

## Supporting information

S1 Table List of ICD-10 codes used in definitions. (DOCX)

S2 Table All-cause mortality rates in the population. (DOCX)
