# Supplementary material for: Cost-effectiveness of influenza vaccine strategies for the elderly in South Korea
Source: PLoS One. 2019 Jan 25;14(1):e0209643. doi: 10.1371/journal.pone.0209643 (PMC6347274; doi:10.1371/journal.pone.0209643)
Supplement: S1 Table — (DOCX) [file pone.0209643.s001.docx]

**S1 Table. List of ICD-10 codes used in definitions**

| Disease Groups | Definition | ICD-10 Codes |
| --- | --- | --- |
| At-risk groups | Respiratory disease | J43, J44, J45, J47, J60–65, J84 |
|  | Heart disease | I05–09, I21–25, I34–37 I42–43, I50 |
|  | Kidney disease | N18–19, I12, I13 |
|  | Liver disease | B18, K70.3, K71.7, K73, K74 |
|  | Neurologic disease | F00–03, G10–14, G20–23, G30–32, G35–37, G40–41, G70–71, G80–83, I60–69, T91.3, R56.8 |
|  | Metabolic disease | E10–14, E15–16, E24 |
|  | Cancer or hematologic disease | C00–97, D46, D55–64, D70–77, D80–89 |
|  | Immunosuppressed state | B20–24, D73 |
| Influenza-like illness diagnostic codes | Acute upper respiratory disease | J00, J01, J02, J03, J04, J05, J06 |
|  | Bronchitis / bronchiolitis | J20, J21, J22 |
|  | Pneumonia | J12, J13, J14, J15, J16, J17.0, J17.1, J17.8, J18 |
|  | Seasonal influenza | J09, 10, 11 |
| Acute complications related influenza | Pneumonia | J12, J13, J14, J15, J16, J17, J18 |
|  | Otitis media | H65, H66, H67 |
|  | Sinusitis | J01, J32 |
|  | Encephalitis | A85.8, A86, A87.8, A87.9, A89, B94.1, G038, G039, G04.0, G04.8, G04.9, G05.1, G05.8, G36 |
|  | Myositis | M60.0, M60.1, M60.8, M60.9 |
|  | Myocarditis/Pericarditis | I41.1, I51.4, I30, I31.9, I40, I51.8, B33.2 |
|  | Acute myocardial infarction | I21, I23, I24 |
|  | Stroke | I63 |
|  | Rhabdomyolysis | M62.8 |
|  | Transverse myelitis | G37.3, G37.8, G37.9 |
| Acute exacerbation of chronic disease | Chronic respiratory disease | J41, J42, J43, J44, J45, J47 |
|  | Chronic liver disease | B18, K70.3, K71.7, K73, K74 |
|  | Chronic kidney disease | N18, N19 |
|  | Chronic heart disease | I25, I42, I50 |
|  | Diabetes | E10-14 |
