# Supplementary material for: Cost-effectiveness of influenza vaccine strategies for the elderly in South Korea
Source: PLoS One. 2019 Jan 25;14(1):e0209643. doi: 10.1371/journal.pone.0209643 (PMC6347274; doi:10.1371/journal.pone.0209643)
Supplement: S2 Table — (DOCX) [file pone.0209643.s002.docx]

**S2 Table. All-cause mortality rates in the population [7]**

| **Age** | Rate | **Age** | Rate |
| --- | --- | --- | --- |
| **65** | 0.00791 | **83** | 0.06643 |
| **66** | 0.00858 | **84** | 0.07466 |
| **67** | 0.00938 | **85** | 0.08377 |
| **68** | 0.01032 | **86** | 0.09374 |
| **69** | 0.01147 | **87** | 0.10459 |
| **70** | 0.01298 | **88** | 0.11636 |
| **71** | 0.01482 | **89** | 0.12909 |
| **72** | 0.01697 | **90** | 0.14279 |
| **73** | 0.01931 | **91** | 0.15747 |
| **74** | 0.02179 | **92** | 0.17313 |
| **75** | 0.02472 | **93** | 0.18976 |
| **76** | 0.02820 | **94** | 0.20733 |
| **77** | 0.03221 | **95** | 0.22580 |
| **78** | 0.03653 | **96** | 0.24511 |
| **79** | 0.04113 | **97** | 0.26517 |
| **80** | 0.04638 | **98** | 0.28590 |
| **81** | 0.05229 | **99** | 0.30716 |
| **82** | 0.05895 | **100** | 1.00000 |
